# Development of key quality indicators for appropriate antibiotic use in the Republic of Korea: results of a modified Delphi survey

**DOI:** 10.1186/s13756-021-00913-y

**Published:** 2021-03-06

**Authors:** Bongyoung Kim, Myung Jin Lee, Se Yoon Park, Song Mi Moon, Kyoung-Ho Song, Tae Hyong Kim, Eu Suk Kim, Hong Bin Kim, Korea Study Group for Antimicrobial Stewardship (KOSGAP)

**Affiliations:** 1 Department of Internal Medicine, Hanyang University College of Medicine, Seoul, Republic of Korea; 2 Department of Internal Medicine, Inje University Sanggye Paik Hospital, Seoul, Republic of Korea; 3 Division of Infectious Diseases, Department of Internal Medicine, Soonchunhyang University Seoul Hospital, Soonchunhyang University College of Medicine, 59, Daesagwan-ro, Yongsan-gu, Seoul, 140-743 Republic of Korea; 4 Department of Internal Medicine, Hallym University Sacred Heart Hospital, Anyang, Republic of Korea; 5 Division of Infectious Diseases, Department of Internal Medicine, Seoul National University Bundang Hospital, Seoul National University College of Medicine, Seongnam, Republic of Korea

**Keywords:** Quality indicator, Anti-bacterial agents, Antibiotic prophylaxis, Bacterial infection

## Abstract

**Background:**

An effective antibiotic stewardship program relies on the measurement of appropriate antibiotic use, on which there is a lack of consensus. We aimed to develop a set of key quality indicators (QIs) for nationwide point surveillance in the Republic of Korea.

**Methods:**

A systematic literature search of PubMed, EMBASE, and Cochrane Library (publications until 20th November 2019) was conducted. Potential key QIs were retrieved from the search and then evaluated by a multidisciplinary expert panel using a RAND-modified Delphi procedure comprising two online surveys and a face-to-face meeting.

**Results:**

The 23 potential key QIs identified from 21 studies were submitted to 25 multidisciplinary expert panels, and 17 key QIs were retained, with a high level of agreement (13 QIs for inpatients, 7 for outpatients, and 3 for surgical prophylaxis). After adding up the importance score and applicability, six key QIs [6 QIs (Q 1–6) for inpatients and 3 (Q 1, 2, and 5) for outpatients] were selected. (1) Prescribe empirical antibiotic therapy according to guideline, (2) change empirical antibiotics to pathogen-directed therapy, (3) obtain culture samples from suspected infection sites, (4) obtain two blood cultures, (5) adapt antibiotic dosage to renal function, and (6) document antibiotic plan. In surgical prophylaxis, the QIs to prescribe antibiotics according to the guideline and initiate antibiotic therapy 1 h before incision were selected.

**Conclusions:**

We identified key QIs to measure the appropriateness of antibiotic therapy to identify targets for improvement and to evaluate the effects of antibiotic stewardship intervention.

## Background

The increased use of broad-spectrum antibiotics is closely related to antimicrobial resistance [1, 2]. Therefore, a need to assess appropriate antibiotic use has been emphasized [3] and includes various steps, such as diagnosis, empirical therapy, dosing, de-escalation, and therapy duration [4]. Moreover, the appropriateness can change with the definition of appropriate antibiotic use [5]. Therefore, researchers have attempted to evaluate antibiotic use's appropriateness with standard criteria by using quality indicators (QIs) developed through expert consensus. In 2018, the European Surveillance of Antimicrobial Consumption group developed QIs to evaluate appropriate antibiotic use in inpatients and outpatients in Europe [6, 7]. In Australia, surveillance of appropriate antibiotic use has been conducted since 2011. Key indicators have been developed [8]; results of appropriate antibiotic use have been generated through national surveillance every year using the key indicators. Nonetheless, it is important to evaluate appropriate antibiotic use with standard criteria, which can then be directly compared between medical institutions and provide feedback to generate suggestions to improve the goals of national antibiotic stewardship. The QIs are diverse, and some indicators are difficult to apply and; therefore, it is necessary to select appropriate QIs through consensus on the antibiotic prescription situation (location), infectious disease, and the type of antibiotics.

In the Republic of Korea (ROK), point surveillance of appropriate antibiotic use was conducted for 20 medical institutions in 2018, and the appropriateness was determined based on expert opinion [9]. This experience generated a need to evaluate appropriate antibiotic use based on specific standards. This study aimed to select potential QIs for appropriate antibiotic use through a systematic literature search and determine the key QIs necessary and important in the ROK through expert consensus. In addition, the developed key QIs will be used in future nationwide point surveillance of appropriate antibiotic use.

## Methods

**Fig. 1:**
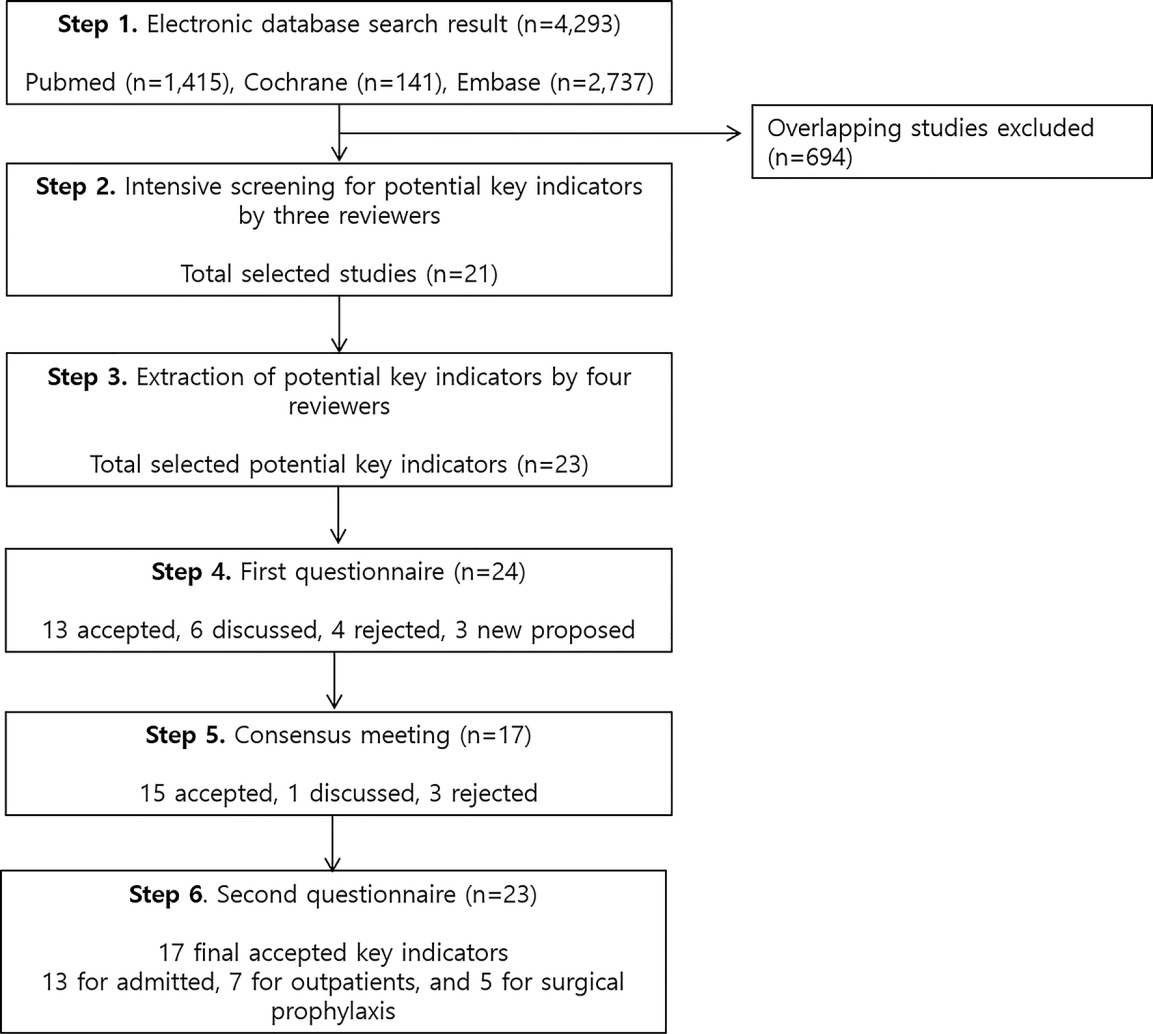
Flowchart of the study procedures

We used a four-step RAND-modified Delphi method to develop a set of QIs to measure the appropriateness of antimicrobial use in adult and hospitalized pediatric patients, outpatients, or patients receiving surgical prophylaxis [10, 11]. Figure 1 presents an overview of the RAND-modified Delphi procedure, which included a comprehensive literature review to develop a list of candidate key QIs, two rounds of an online survey, and a face-to-face meeting with the panelists. The consensus procedure combined the individual opinions of multidisciplinary expert panels. All the panel members consented to participate in the study and were aware that their answers would be used for research.

### Systematic search for generating key QIs

We performed a systematic search using a protocol designed by two independent medical librarians (D.W.S and M.L). We screened the literature using databases of PubMed, EMBASE, and Cochrane Library for papers published up to 20th November 2019. The search strategy is shown in Additioinal file 1: Fig. S1 and was directed towards identifying evidence-based QIs for antibiotic use (e.g., literature review or evidence-based guideline). Papers written in English and discussed using systemically administered antibiotic drugs in inpatients, outpatients, and surgical prophylaxis were included, except for case reports. This study aimed to develop key QIs useful to assess the antibiotic use appropriateness in treating all bacterial infections and surgical prophylaxis. Therefore, we excluded antiviral, antifungal, antiparasitic, or antituberculosis drugs. Using EndNote software (version X7.1, 2020 Clarivate), two researchers (B.K. and S.Y.P.) independently examined all titles and abstracts to select papers that described QIs. Any disagreement on the inclusion or exclusion of studies was resolved through discussion with a third author (S.M.). If no abstract was available or there was a lack of information for the eligibility assessment, papers were selected for full-text screening. The inclusion/exclusion criteria evaluation for full-text screening was performed by two researchers (B.K. and S.Y.P.).

### Selection of potential key QIs

Data on potential QIs were extracted by four researchers (B.K., M.J.L., S.Y.P., and S.M.). The QIs were excluded if they were: not concerned with antibiotic use for a specific group of patients, non-normative, or developed for each institution and not for patients. The extracted QIs were then clustered into different non-overlapping logical themes based on the definition of responsible use. When a QI could be allocated to more than one theme, the predominant theme was chosen based on consensus between two authors (B.K. and S.Y.P.). Duplicates were removed, and the QIs were rephrased as a recommendation. The clustering, aggregating, and rephrasing steps were undertaken consensually among four authors (B.K., M.J.L., S.Y.P., and S.M.).

### First online survey

We emailed invitations to different specialists for their participation in 25 expert panels. The panel of doctors comprised experts working at university-affiliated hospitals in the ROK. During panel selection, we aimed to select experts representative and responsible for antibiotic prescriptions. The panel comprised infectious disease specialists (n = 13), laboratory medicine doctors (n = 3), pediatric infectious diseases specialists (n = 2), urologists (n = 2), otorhinolaryngologist (n = 1), gastroenterologist (n = 1), pulmonologist (n = 1), general surgeon (n = 1), and researchers of the National Evidence-Based Healthcare Collaborating Agency (n = 1) (Additioinal file 3: Table S2). To rate the degree to which the potential QI described appropriate antibiotic use, a Likert scale, ranging from 1 (‘definetly inappropriate care’) to 7 (‘definitely appropriate care’), was used. The panelists could rephrase the potential indicator and could even add new items. A consensus was defined as the case wherein ≥ 70% of the scores were in the top quartile (scores 6 and 7); similarly, scores with < 70% agreement were defined as disagreement. QIs with a median score of 6 or 7 were accepted if there was agreement. If there was disagreement and the median score was ≤ 5, the QI was rejected. Thus, QIs with a median score of 6 and 7 with disagreement were discussed during the expert panel meeting. In addition, we graded each QI using a Likert scale score, ranging from 1 to 7, as relevant to inpatient care, outpatient care, or surgical prophylaxis. If the score was 6 or 7, we considered it to be an appropriate QI.

### Expert panel meeting

All panel members were invited to a face-to-face panel meeting. Before the meeting, all participants received a personal feedback report with the results of the first online questionnaire. The agenda of the panel meeting was to present the results of the first round of the survey and to discuss the QIs with a median score of 6 or 7 that had inadequate consensus. These QIs were accepted if at least 70% of the experts concurred. In addition, newly added potential QIs were discussed, and the accepted QIs with comments from the experts were rephrased base on consensus.

### Second online survey

A second questionnaire that included all the selected and rephrased QIs were sent with a personal feedback report (providing the results of the previous two steps of the consensus procedure) to all participating panelists. The panelists were asked to select from the following three answers: ‘agree,’ ‘disagree,’ and ‘cannot assess.’ The rephrased indicators were accepted if at least 70% of the experts agreed with the new formulation. Furthermore, we asked the panelists to consider the importance of potential QIs that could be used as key indicators for antibiotic use in the ROK, with a Likert scale score from 1 ‘less important’ to 5 ‘highest importance’. If the QIs could be evaluated in point surveillance, we considered them applicable. This was finally confirmed by four researchers (B.K., M.J.L., S.Y.P., and S.M.). We excluded QIs with durations in which appropriateness was difficult to evaluate by point surveillance.

## Results

### Literature search and selection of potential key QIs

The systematic literature search identified 4293 articles, of which only 21 articles were finally selected after screening against the inclusion/exclusion criteria (Additioinal file 2: Table S1) [6, 7, 12–29]. From these 21 articles, we initially derived 192 QIs. After duplications were removed and the quantitative or institution-level QIs were excluded, 23 QIs were finally included (Table 1). This list of 23 QIs was presented to the panelists in the first round of the consensus procedure.

**Table 1:** Final lists of potential key quality indicators

Nos.	Potential key quality indicators
QI 1	Empirical systemic antibiotic therapy should be prescribed according to the institutional, national, or international guideline
QI 2	Before starting systemic antibiotic therapy, at least two sets of blood cultures should be taken
QI 3	When starting systematic antibiotic therapy, specimens for culture from suspected sites of infection should be taken as soon as possible, preferably before antibiotics are started
QI 4	An antibiotic plan should be documented in the case notes at the start of systemic antibiotic therapy
QI 5	Systemic antibiotic therapy should be switched from i.v. to oral antibiotic therapy within 48–72 h on the basis of the clinical condition and when oral treatment is adequate
QI 6	Empirical antibiotic therapy should be changed to pathogen-directed therapy if culture results become available
QI 7	Dose and dosing interval of systemic antibiotic therapy should be adapted to renal function
QI 8	Therapeutic drug monitoring should be performed when the therapy duration is > 3 days for aminoglycosides and > 5 days for vancomycin
QI 9	Empirical antibiotic therapy for presumed bacterial infection should be discontinued based on the lack of clinical and/or microbiological evidence of infection. The maximum duration of empirical systemic antibiotic treatment should be 7 days
QI 10	Surgical prophylaxis antibiotic therapy should be prescribed according to guideline
QI 11	Surgical prophylaxis antibiotic therapy should be initiated within 1 h before incision
QI 12	Surgical prophylaxis antibiotic therapy should be discontinued within 1 day
QI 13	Antibiotic therapy in adult patients with sepsis should be started intravenously
QI 14	Contraindications (history of allergy, anaphylaxis, or toxicity) should be taken into account when prescribing antibiotics
QI 15	Acute upper respiratory infections and bronchitis should not be treated with antibiotics within the first 3 days, unless there is documented indication for treatment
QI 16	Antibiotics should be prescribed within appropriate cost
QI 17	Antibiotics should be prescribed in appropriate duration
QI 18	Antibiotics with anaerobic activity combination of two or more antimicrobials
QI 19	Outpatients with acute tonsillitis/pharyngitis should undergo a group A streptococcal diagnostic test to decide whether or not they should receive antibiotics
QI 20	Follow up cultures 4–7 days after initial blood culture positivity (bloodstream infection due to *Staphylococcus aureus* and fungi)
QI 21	Documented significant single-organism bacteriuria
QI 22	Perform ID specialist bedside consultation in hospitalized patient
QI 23	Appropriate pharmacokinetics

These potential quality indicators are final version after Delphi procedure

*QI* quality indicator

### First online survey

The first online round of the survey was conducted from 27th December 2019 to 8th January 2020. In total, 24 (96%, 24/25) panelists responded to the first online questionnaire. Among the 23 potential QIs, 13 QIs (57%) were accepted, 6 (26%) were discussed, and 4 (17%) were rejected (Table 2). Three additional QIs were suggested from the results of the online survey: (1) dose adjustment of surgical antibiotic prophylaxis according to body weight, (2) Percentage of blood cultures among those who received systemic antibiotic use, and (3) empirical antibiotic therapy should be prescribed in community-acquired or healthcare-associated infections. The application of QIs was appropriate in hospitalized patients (n = 17), outpatients (n = 9), and surgical prophylaxis (n = 6).

**Table 2:** Result of the consensus procedure

Quality indicator	First questionnaire			Application			Consensus meeting	Second questionnaire		
	Median	% in highest quartile	Conclusion	Admitted	Outpatient	Surgical prophylaxis		Agreement (%)	Total score	Conclusion
1	7	75	Accepted	Yes	Yes	Yes	Rephrased	100	114	Accepted
2	6	75	Accepted	Yes	No	No	Rephrased	100	100	Accepted
3	7	87.5	Accepted	Yes	No	No	Rephrased	100	103	Accepted
4	6	54.5	Discussed	Yes	No	No	Rephrased, accepted	91	89	Accepted
5	6	62.5	Discussed	Yes	No	No	Rephrased, accepted	87	82	Accepted
6	7	83.3	Accepted	Yes	Yes	No		NA	109	Accepted
7	7	79.2	Accepted	Yes	Yes	No		NA	96	Accepted
8	6	62.5	Discussed	Yes	No	No	Accepted	70	52	Accepted
9	6	70.8	Accepted	Yes	Yes	No		91	88	Accepted
10	7	95.8	Accepted	No	No	Yes	Merge into QI 1	NA	113	
11	7	91.7	Accepted	No	No	Yes		NA	109	Accepted
12	6.5	79.2	Accepted	No	No	Yes		NA	103	Accepted
13	6	62.5	Discussed	Yes	No	No	Rejected	NA	NA	Rejected
14	7	87.5	Accepted	Yes	Yes	No		87	87	Accepted
15	6	58.3	Discussed	Yes	Yes	No	Rejected	NA	NA	Rejected
16	4	12.5	Rejected	No	No	No		NA	NA	Rejected
17	6	87.5	Accepted	Yes	Yes	Yes		NA	92	Accepted
18	6.5	75	Accepted	Yes	Yes	No	Rephrased	91	84	Accepted
19	5	41.7	Rejected	No	No	No		NA	NA	Rejected
20	6	75	Accepted	Yes	No	No	Rephrased	78	69	Accepted
21	5	41.7	Rejected	Yes	No	No		NA	NA	Rejected
22	5	41.7	Rejected	Yes	No	No		NA	NA	Rejected
23	6	58.3	Discussed	No	No	No	Rejected	NA	NA	Rejected
24			Added	No	No	No	Accepted	74	58	Accepted
25			Added	No	No	Yes	Merge into QI 1	NA	NA	
26			Added	Yes	Yes	No	Merge into QI 2	NA	NA	

*NA* not applicable, *Q* quality indicator

### Expert panel meeting

The face-to-face panel meeting was held on 10th January 2020, and 16 (64%, 16/25) panelists participated in this meeting. Among the six potential QIs from the first online questionnaire round discussed, three were conclusively accepted, and three were rejected. QI 10 was merged with QI 1. Among the three suggested QIs from the first survey, one QI (dose adjustment of surgical antibiotic prophylaxis according to body weight) was accepted. The other two QIs (percentage of blood culture among those with systemic antibiotic use and empirical antibiotic therapy should be prescribed in community-acquired or healthcare-associated infections) were merged with QI 1 and QI 2, respectively (Table 2).

### Second online survey

A total of 17 potential QIs (16 pre-existing QIs, and one new QI was added) were included in the second online questionnaire. Twenty-three (92%, 23/25) panelists participated in this survey. All potential QIs were accepted (Table 2). The ranking of the final selected key QIs and applicability in the point surveillance study is shown in Table 3 (inpatients and out-patients) and Table 4 (surgical prophylaxis). Based on the sum of the importance score and applicability, six key QIs for hospitalized patients and three (following number 1–3) for outpatients were finally selected. (1) Prescribe empirical antibiotic therapy according to the guideline, (2) change empirical antibiotic to pathogen-directed therapy, (3) obtain culture samples from suspected sites of infection, (4) take two blood cultures, (5) adapt antibiotic dosage to renal function, and (6) document the antibiotic plan. Concerning surgical prophylaxis, the recommendations to prescribe according to the guideline and initiate antibiotics 1 h before the incision were finally selected.

**Table 3:** Ranking of final selected key quality indicators for admitted and outpatients and applicability in point surveillance study

Quality indicators	Inpatients			Outpatients		
	Ranking	Total score	Applicability	Ranking	Total score	Applicability
QI 1. Empirical systemic antibiotic therapy should be prescribed according to the institutional, national, or international guideline	1	114	Yes	1	114	Yes
QI 6. Empirical antibiotic therapy should be changed to pathogen-directed therapy if culture results become available	2	109	Yes	2	109	Yes
QI 3. When starting systematic antibiotic therapy, specimens for culture from suspected sites of infection should be taken as soon as possible, preferably before antibiotics are started	3	103	Yes			
QI 2. Before starting systemic antibiotic therapy, at least two sets of blood cultures should be taken	4	100	Yes			
QI 7. Dose and dosing interval of systemic antibiotic therapy should be adapted to renal function	5	96	Yes	3	96	Yes
QI 17. Antibiotics should be prescribed in appropriate duration	6	92	No	4	92	No
QI 4. An antibiotic plan should be documented in the case notes at the start of systemic antibiotic therapy	7	89	Yes			
QI 9. Empirical antibiotic therapy for presumed bacterial infection should be discontinued based on the lack of clinical and/or microbiological evidence of infection. The maximum duration of empirical systemic antibiotic treatment should be 7 days	8	88	No	5	88	No
QI 14. Contraindications (history of allergy, anaphylaxis, or toxicity) should be taken into account when prescribing antibiotics	9	87	No	6	87	No
QI 18. Antibiotics with anaerobic activity combination of two or more antimicrobials	10	84	No			
QI 5. Systemic antibiotic therapy should be switched from i.v. to oral antibiotic therapy within 48–72 h on the basis of the clinical condition and when oral treatment is adequate	11	82	No			
QI 20. Follow up cultures 4–7 days after initial blood culture positivity (bloodstream infection due to *Staphylococcus aureus* and fungi)	12	69	No			
QI 8. Therapeutic drug monitoring should be performed when the therapy duration is > 3 days for aminoglycosides and > 5 days for vancomycin	13	52	No	7	84	No

*QI* quality indicator

**Table 4:** Ranking of final selected key quality indicators for surgical prophylaxis and applicability in point surveillance study

Ranking	Quality indicators	Total score	Applicability
1	QI 10. Surgical prophylaxis antibiotic therapy should be prescribed according to guideline	113	Yes
2	QI 11. Surgical prophylaxis antibiotic therapy should be initiated within 1 h before incision	109	Yes
3	QI 12. Surgical prophylaxis antibiotic therapy should be discontinued within 1 day	103	No
4	QI 17. Antibiotics should be prescribed in appropriate duration	92	No
5	QI 24. Dose of surgical prophylaxis antibiotics should be adjusted according to body weight	58	No

*QI* quality indicator

## Discussion

We selected key QIs from among the potential QIs extracted in a systematic literature search followed by a RAND-modified Delphi survey procedure. These key QIs are intended to be universally applicable, regardless of the type of infectious disease, location, or situation of antibiotic use. This study is practical in that the key QIs can be applied in all antibiotic use situations, including inpatients, outpatients, and surgical prophylaxis, and were selected through an expert panel consensus with ranking for importance and consideration of applicability in point surveillance. The selected key QIs for hospitalized patients and outpatients should be applied in a point surveillance study for antibiotic appropriateness in 75 hospitals in the ROK in 2020.

Finally, selected Key QIs were included in several steps of antibiotic prescription, such as diagnosis (QI 2, 3, and 4), empiric therapy (QI 1), dosing (QI 7), and definite therapy (QI 6). QIs related to therapy duration were not selected, although it got a considerably high score on the importance scale among experts. Therapy duration is increasingly an important focus area for antimicrobial stewardship activity. However, expected applicability was below 10% based on previous studies [9, 13, 22]. Therefore, we excluded these QIs in the final selection. If point surveillance or applicability is not considered, the QIs selected in the second online survey can be used by an antibiotic stewardship team or a researcher, depending on the surveillance purpose.

Assessing antibiotic use's appropriateness can be divided mainly into evaluating the entire antibiotic options and evaluating certain antibiotics or diseases [30]. In addition, the scope of assessment varies greatly depending on the study, from empirical antibiotics to therapeutic antibiotics, dosing, narrow-spectrum antibiotics, duration of treatment, and presence of medical records [4]. Moreover, the methods of evaluation can be divided into judgments based on guidelines, judgment by experts, and judgments using QIs [5]. Therefore, the scope of evaluation and the criteria for assessing appropriate antibiotic use should be determined by researchers according to the surveillance purpose. Because of the increasing need for antibiotic stewardship, national surveillance of appropriate antibiotic use commenced in 2018 in the ROK. To establish a national target of antibiotic stewardship, we included the entire gamut of antibiotic use, regardless of infectious disease type.

To date, the most widely used QIs are 11 indicators that were selected by van den Bosch et al. [14] in the Netherlands. These QIs were selected from 1574 studies through a literature review. The RAND-modified Delphi procedure was used to propose the final 11 QIs; of these, nine were proposed at the patient level and two at the institutional level [14]. Those 11 QIs were applied to 1890 inpatients in 22 hospitals in the Netherlands in 2011 and 2012. The applicability of three QIs (therapeutic drug monitoring, adapting antibiotics to renal function, discontinuation of empirical therapy in case of a lack of clinical and/or microbiological evidence of infection) were less than 10% [13]. In this study, the nine potential key QIs of patient-level by van den Bosch et al. were merged and finally selected after obtaining consensus. Considering their applicability in the point surveillance study, we finally excluded key QIs on duration, such as “appropriate duration” and “discontinue antibiotic therapy if an infection is not confirmed.” The “adapted dose and dosing interval of antibiotics to renal function” QI was expected to have low applicability. However, it was finally accepted because this key QI can be evaluated by point surveillance, and the panel considered it important.

The advantage of QIs over expert judgment is that the evaluation results of each QI show the areas that need improvement. In addition, comparisons with other medical institutions show whether the specified institution needs improvement. Another advantage is that the same criteria can be applied every year to determine whether one’s institution has improved. In Australia, four key QIs (documentation of indication, documentation of review or stop date, surgical prophylaxis for > 24 h. and noncompliance with guidelines) and inappropriate antibiotic prescription rates are presented annually along with the target levels [8]. Based on the collected data, the researchers could identify priority areas for targeted quality improvement initiatives directed toward antimicrobial prescribing patterns. In comparison, judgments may differ among experts, and it is difficult to ascertain the aspects required for improvement. Thus, efforts to select the key QIs agreed upon by each country or institution are a necessary task in the future.

A limitation of this study is that members of the expert panel were only Koreans, and none was with pharmacy expertise. The QIs would be difficult to apply in other countries with different medical environments from the ROK. Pharmacy expertise was suggested as a core element of hospital antibiotic stewardship programs; however, the current antimicrobial stewardship program is mainly performed by infectious diseases specialists in the ROK [31, 32]. Second, the literature search scope was restricted to English-only papers and limited to only a literature database, without a website search. Therefore, we may have missed important papers in other languages. However, English is considered a major global medium in the scientific literature directed toward a global audience.

## Conclusions

In this study, key QIs that can be applied to hospitalized patients, outpatients, and surgical prophylaxis were selected in the ROK. Not only will these key QIs be applied in national point surveillance initiatives in the ROK in 2020, but also other users will be able to use the QIs for antibiotic stewardship programs or in educational materials for appropriate antibiotic use. Evaluators will be able to prioritize antibiotic use based on the important indicators identified in this study. It is expected that the application of key QIs will complement and apply the advantages and limitations of the evaluation indicators experienced by the actual evaluators and researchers.

## Supplementary Information

**Additional file 1. Figure**: Search strategy for the literature review.

**Additional file 2. Table 1**: Final list of 21 articles selected after a systematic literature search.

**Additional file 3. Table 2**: Title and affiliations of expert panels.

## Data Availability

The datasets used and/or analyzed during the current study are available from the corresponding author on reasonable request.

## Abbreviations

QI: Quality indicators
ROK: Republic of Korea
