# Supplementary material for: Development of key quality indicators for appropriate antibiotic use in the Republic of Korea: results of a modified Delphi survey
Source: Antimicrob Resist Infect Control. 2021 Mar 6;10:48. doi: 10.1186/s13756-021-00913-y (PMC7937201; doi:10.1186/s13756-021-00913-y)
Supplement: Supplementary file 1 — Additional file 1. Figure: Search strategy for the literature review. [file 13756_2021_913_MOESM1_ESM.pptx]

## Slide 1
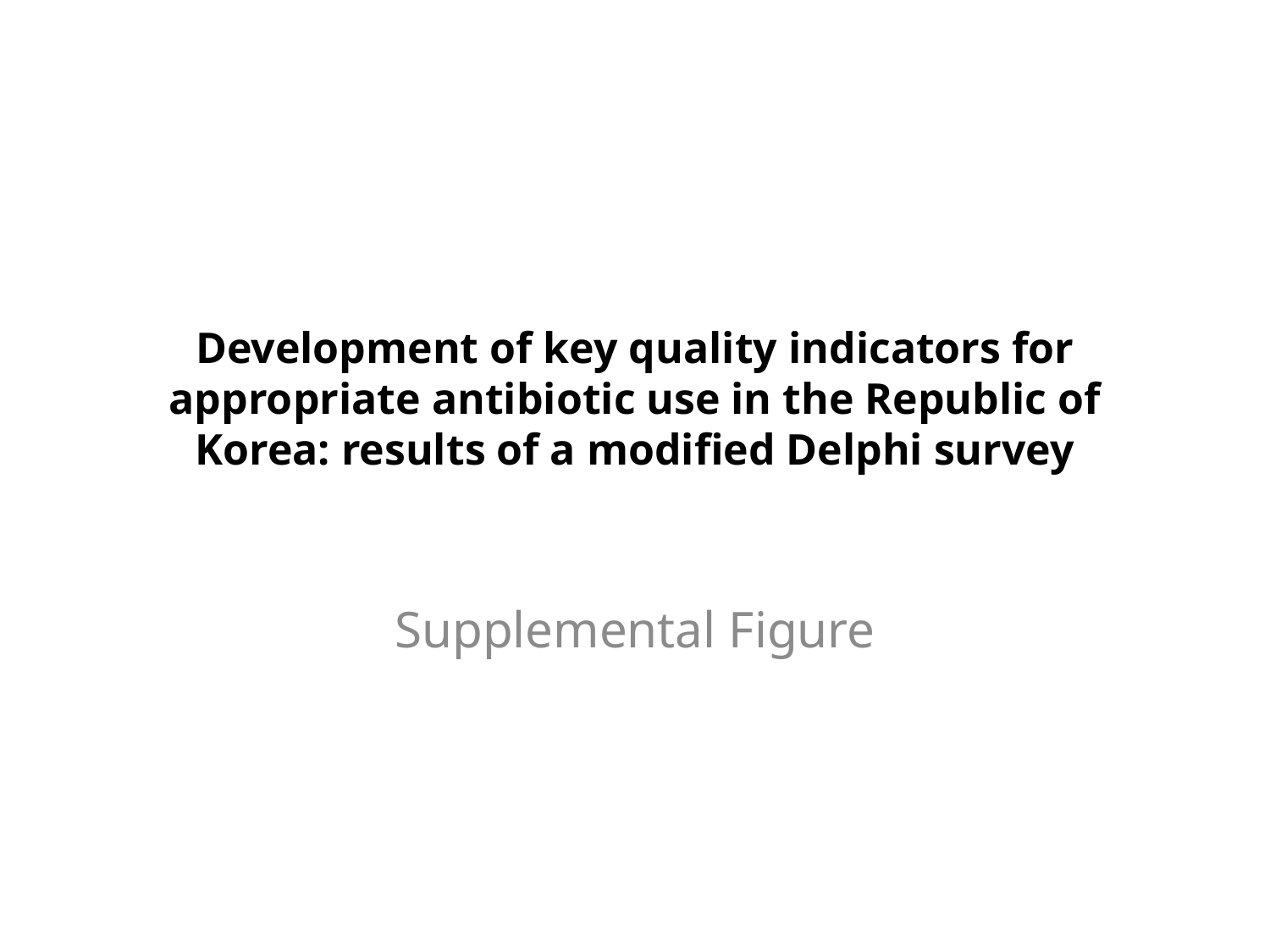

# Development of key quality indicators for appropriate antibiotic use in the Republic of Korea: results of a modified Delphi survey
Supplemental Figure

## Slide 2
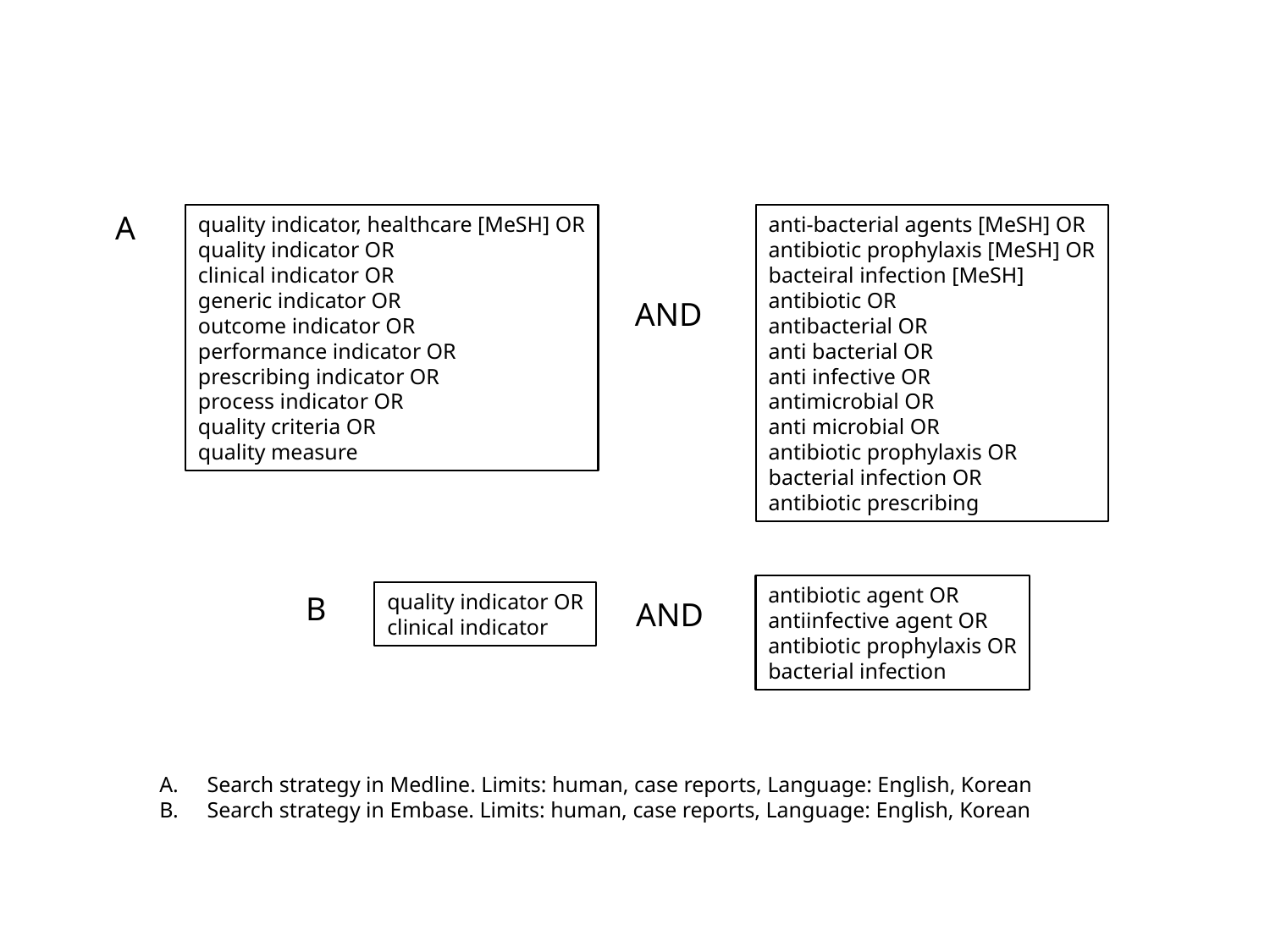

A
quality indicator, healthcare [MeSH] OR
quality indicator OR
clinical indicator OR
generic indicator OR
outcome indicator OR
performance indicator OR
prescribing indicator OR
process indicator OR
quality criteria OR
quality measure
anti-bacterial agents [MeSH] OR
antibiotic prophylaxis [MeSH] OR
bacteiral infection [MeSH]
antibiotic OR
antibacterial OR
anti bacterial OR
anti infective OR
antimicrobial OR
anti microbial OR
antibiotic prophylaxis OR
bacterial infection OR
antibiotic prescribing
AND
antibiotic agent OR
antiinfective agent OR
antibiotic prophylaxis OR
bacterial infection
B
quality indicator OR
clinical indicator
AND
Search strategy in Medline. Limits: human, case reports, Language: English, Korean
Search strategy in Embase. Limits: human, case reports, Language: English, Korean
