# Supplementary material for: Development of key quality indicators for appropriate antibiotic use in the Republic of Korea: results of a modified Delphi survey
Source: Antimicrob Resist Infect Control. 2021 Mar 6;10:48. doi: 10.1186/s13756-021-00913-y (PMC7937201; doi:10.1186/s13756-021-00913-y)
Supplement: Supplementary file 2 — Additional file 2. Table 1: Final list of 21 articles selected after a systematic literature search. [file 13756_2021_913_MOESM2_ESM.docx]

**Supplemental Table 1.** Final list of 21 articles that were selected after a systematic literature search.

| First author | Title | Reference |
| --- | --- | --- |
| Van der Velden AW, et al. | Usefulness of quality indicators for antibiotic use: case study for the Netherlands. | Int J Qual Health Care. 2016;28:838-842. |
| Van den Bosch CMA, et al. | Applicability of generic quality indicators for appropriate antibiotic use in daily hospital practice: a cross-sectional point-prevalence multicenter study. | Clin Microbiol Infect. 2016;22:888.e881-888.e889. |
| Van den Bosch CMA, et al. | Quality indicators to measure appropriate antibiotic use in hospitalized adults | Clin Infect Dis. 2015;60:281-291. |
| Van Daalen FV, et al. | A cluster randomized trial for the implementation of an antibiotic checklist based on validated quality indicators: the AB-checklist | BMC Infect Dis. 2015;15:134. |
| Van Daalen, et al. | Effect of an antibiotic checklist on length of hospital stay and appropriate antibiotic use in adult patients treated with intravenous antibiotics: a stepped wedge cluster randomized trial | Clin Microbiol Infect. 2017;23:485.e481-485.e488. |
| Thern J, et al. | Selection of hospital antimicrobial prescribing quality indicators: a consensus among German antibiotic stewardship (ABS) networkers | Infection. 2014;42:351-362. |
| Schmitt C, et al. | Applying validated quality indicators to surgical antibiotic prophylaxis in a Brazilian hospital: Learning what should be learned | Am J Infect Control. 2012;40:960-962. |
| Monnier AA, et al. | Quality indicators for responsible antibiotic use in the inpatient setting: A systematic review followed by an international multidisciplinary consensus procedure | J Antimicrob Chemother. 2018;73:vi30-vi39. |
| Le Marechal M, et al. | Quality indicators assessing antibiotic use in the outpatient setting: A systematic review followed by an international multidisciplinary consensus procedure | Antimicrob Chemother. 2018;73:vi40-vi49. |
| Kallen MC, et al. | A systematic review of quality indicators for appropriate antibiotic use in hospitalized adult patient | Infect Dis Rep. 2017;9(1):6821. |
| Coenen S, et al. | European Surveillance of Antimicrobial Consumption (ESAC): quality indicators for outpatient antibiotic use in Europe | Qual Saf Health Care. 2007;16:440-445. |
| Berrevoets MAH, et al. | Monitoring, documenting and reporting the quality of antibiotic use in the Netherlands: A pilot study to establish a national antimicrobial stewardship registry | BMC Infect Dis. 2017;17:565 |
| Arcenillas P, et al. | Assessment of Quality Indicators for Appropriate Antibiotic Use | Antimicrob Agents Chemother. 2018 Nov 26;62(12):e00875-18. |
| Adriaenssens N, et al. | European Surveillance of Antimicrobial Consumption (ESAC): disease-specific quality indicators for outpatient antibiotic prescribing | BMJ Qual Saf 2011 Sep;20(9):764-772. |
| Kallen M, et al. | Development of actionable quality indicators and an action implementation toolbox for appropriate antibiotic use at intensive care units: A modified-RAND Delphi study | PLoS One. 2018;13:e0207991. |
| Howard P, et al. | ESGAP inventory of target indicators assessing antibiotic  prescriptions: a cross-sectional survey | J Antimicrob Chemother. 2017;72:2910-2914. |
| van den Bosch et al, et al. | Appropriate antibiotic use reduces length of hospital stay | J Antimicrob Chemother. 2017;72:923-932. |
| Zarb P, et al | Identification of targets for quality improvement in antimicrobial prescribing: the web-based ESAC Point Prevalence Survey 2009 | J Antimicrob Chemother. 2011;66:443-449. |
| van Daalen F, et al | Implementation of an antibiotic checklist increased appropriate antibiotic use in the hospital on Aruba | Int J Infect Dis. 2017;59:14-21. |
| Berrevoets M, et al | Monitoring, documenting and reporting the quality of antibiotic use in the Netherlands: a pilot study to establish a national antimicrobial stewardship registry | BMC Infect Dis. 2017;17:565. |
| Gyssens I, et al | Quality measures of antimicrobial drug use | Int J Antimicrob Agents. 2001;17:9-19. |
