# Supplementary material for: Development of key quality indicators for appropriate antibiotic use in the Republic of Korea: results of a modified Delphi survey
Source: Antimicrob Resist Infect Control. 2021 Mar 6;10:48. doi: 10.1186/s13756-021-00913-y (PMC7937201; doi:10.1186/s13756-021-00913-y)
Supplement: Supplementary file 3 — Additional file 3. Table 2: Title and affiliations of expert panels. [file 13756_2021_913_MOESM3_ESM.docx]

**Supplemental Table 2.** Title and affiliations of expert panels

| **Name** | **Title(s)** | **Affiliation(s)** |
| --- | --- | --- |
| Bongyoung Kim, MD, PhD | Adult Infectious Diseases Physician | Hanyang University Seoul Hospital |
| Eu Suk Kim, MD, PhD | Adult Infectious Diseases Physician | Seoul National Bundang Hospital |
| Tae Hyong Kim, MD, PhD | Adult Infectious Diseases Physician | Soonchunhyang University Seoul Hospital |
| Hong-Bin Kim, MD, PhD | Adult Infectious Diseases Physician | Seoul National Bundang Hospital |
| Song Mi Moon, MD, PhD | Adult Infectious Diseases Physician | Hallym University Sacred Heart Hospital |
| Kyung-Ho Song, MD, PhD | Adult Infectious Diseases Physician | Seoul National Bundang Hospital |
| Se Yoon Park, MD, PhD | Adult Infectious Diseases Physician | Soonchunhyang University Seoul Hospital |
| Yoon Soo Park, MD, PhD | Adult Infectious Diseases Physician | Yongin Severance Hospital |
| Joon Young Song, MD, PhD | Adult Infectious Diseases Physician | Korea University Guro Hospital |
| Myung Jin Lee, MD, MS | Adult Infectious Diseases Physician | Inje University Sanggye Paik Hospital |
| Mi Suk Lee, MD, PhD | Adult Infectious Diseases Physician | Kyung Hee University Hospital |
| Hyung-Ha Chang, MD, PhD | Adult Infectious Diseases Physician | Kyungpook Natinal University Hospital |
| Su-Mi Choi, MD, PhD | Adult Infectious Diseases Physician | Yeouido St. Mary’s Hospital, The Catholic University of Korea |
| Ji Young Park, MD, PhD | Pediatric Infectious Diseases Doctor | ChungAng University Hospital |
| Hyunju Lee, MD, PhD | Pediatric Infectious Diseases Doctor | Seoul National Bundang Hospital |
| Hyuk Min Lee, MD, PhD | Clinical Pathologist | Gangnam Severance Hospital |
| Ki-Ho Hong, MD, PhD | Clinical Pathologist | Seoul Medical Center |
| Jeong Su Park, MD, PhD | Clinical Pathologist | Soonchunhyang University Seoul Hospital |
| Jae Wook Kim, MD, PhD | Otolartyngology Specialist | Soonchunhyang University Seoul Hospital |
| Seung-Ju Lee, MD, PhD | Urologist | St. Vincent’s Hospital, The Catholic University of Korea |
| U-Syn Ha, MD, PhD | Urologist | Seoul St. Mary’s Hospital, The Catholic University of Korea |
| Jee Youn Oh, MD, PhD | Pulmonologist | Korea University Guro Hospital |
| Jaihwan Kim, MD, PhD | Gastroenterologits | Seoul National Bundang Hospital |
| Kil Yeon Lee, MD, PhD | General Surgeon | Kyung Hee University Hospital |
| Dong-A Park | National Evidence-Based Healthcare Collaborating Agency | Researcher |
